# Late Positivity Does Not Meet the Criteria to be Considered a Proper Neural Correlate of Perceptual Awareness

**DOI:** 10.3389/fnsys.2020.00036

**Published:** 2020-07-07

**Authors:** Chiara Mazzi, Gaetano Mazzeo, Silvia Savazzi

**Affiliations:** Perception and Awareness (PandA) Laboratory, Department of Neuroscience, Biomedicine and Movement Sciences, University of Verona, Verona, Italy

**Keywords:** neural correlates of consciousness (NCC), perceptual awareness, event-related potentials (ERP), EEG, contrastive analysis, visual awareness negativity (VAN), late positivity (LP), response criterion

## Abstract

Contrastive analysis has been widely employed in the search for the electrophysiological neural correlates of consciousness. However, despite its clear logic, it has been argued that it may not succeed in isolating neural processes solely involved in the emergence of perceptual awareness. In fact, data from contrastive analysis would be contaminated by potential confounding factors reflecting distinct, though related, processes either preceding or following the conscious perception. At present, the ERP components representing the proper correlates of perceptual awareness still remain to be identified among those correlating with awareness (i.e., Visual Awareness Negativity, VAN and Late Positivity, LP). In order to dissociate visual awareness from post-perceptual confounds specifically related to decision making, we manipulated the response criterion, which affects how a percept is translated into a decision. In particular, while performing an orientation discrimination task, participants were asked to shift their response criterion across sessions. As a consequence, the resulting modulation should concern the ERP component(s) not exclusively reflecting mechanisms regulating the subjective conscious experience itself but rather the processes accompanying it. Electrophysiological results showed that N1 and P3 were sensitive to the response criterion adopted by participants. Additionally, the more the participants shifted their response criterion, the bigger the ERP modulation was; this was consequently indicative of the critical role of these components in the decision-making processes regardless of awareness level. When considering data independently from the response criterion, the aware vs. unaware contrast showed that both VAN and LP were significant. Crucially, the LP component was also modulated by the interaction of awareness and response criterion, while VAN results to be unaffected. In agreement with previous literature, these findings provide evidence supporting the hypothesis that VAN tracks the emergence of visual awareness by encoding the conscious percept, whereas LP reflects the contribution from post-perceptual processes related to response requirements. This excludes a direct functional role of this later component in giving rise to perceptual awareness.

## Introduction

Neural correlates of consciousness (hereafter called NCCs), according to an accepted operationalization, are defined as the minimal subset of neural activations sufficient to trigger a conscious experience (Chalmers, 2000; Crick and Koch, 2003; Fink, 2016). A considerable amount of EEG studies devoted to disclosing the temporal dimension of NCCs has identified two possible candidates for electrophysiological markers of perceptual conscious experience: the Visual Awareness Negativity (VAN), occurring at occipito-temporal sites in the N2 latency range, and the Late Positivity (LP), which is maximally distributed over centro-parietal sites in the P3 time window (at about 300–500 ms after the stimulus onset; Koivisto and Revonsuo, 2003, 2010). These components, though opposite in polarity, are both characterized by a greater amplitude for aware than unaware condition and for this reason are computed as difference waves of the N2 and P3 components. While VAN has been associated with perceptual awareness quite recently and attempts to localize its source are still sparse, a study published in 1971 already reported a larger P3 amplitude in response to detected near-threshold auditory compared to the missed ones (Hillyard et al., 1971). So far, P3 has been shown to be modulated by a variety of factors and observed across multiple experimental tasks. Therefore, different cognitive mechanisms have been ascribed to this component family, and its generator is still a matter of extensive debate (Polich, 2007; Volpe et al., 2007; Verleger, 2020).

The two components were principally pinpointed by means of the so-called “contrastive analysis” (Baars, 1988), which, in perceptual awareness literature, has been applied to several paradigms, such as simple visual detection or discrimination tasks, backward masking, binocular rivalry, or inattentional blindness (for a review see Kim and Blake, 2005), and combined with brain imaging techniques of a different nature. According to this approach, the physical properties of near-threshold visual stimuli are kept constant across repeated trials, while conscious experience fluctuates as a result of experimental manipulation. Then, aware and unaware trials are *post hoc* sorted on the basis of participants’ subjective reports and contrasted against each other.

Critically, the contrastive approach may have led to an oversimplification. Indeed, despite its clear logic, this approach does not take into account any cognitive process systematically occurring in association with perceptual awareness. As such, the contrastive method may not succeed in segregating the solely “proper” NCCs for the reason that the contrasted conditions would not exclusively differ in terms of awareness. Results, indeed, would include related, but conceptually distinct, processes either preceding or following the actual conscious perception (Melloni et al., 2011; Aru et al., 2012; Pitts et al., 2014; Sandberg et al., 2014).

Specifically, the function of pre-conscious processes is ensuring the occurrence and/or boosting the subsequent perceptual experience. Reduced pre-stimulus alpha oscillations responsible for increased cortical excitability, a certain level of arousal, and, in general, attention-based processes represent possible examples of awareness prerequisites that can selectively enhance relevant processing and/or inhibit irrelevant ones. Conversely, post-perceptual processes involve, among others, working memory and decision-making aspects, allowing the access and the maintenance of the perceptual information needed for a decision. This represents a critical issue since contrastive experimental designs mostly rely upon trial-by-trial subjective reports. As exemplified by the analogy of the “refrigerator door problem” reported in Pitts et al. (2014), if you are interested in identifying the minimal sufficient conditions (i.e., neural correlates of consciousness) under which the refrigerator lights up (where the light represents the conscious percept), you necessarily need to open the door (i.e., asking observers to openly rate their conscious experience). However, we cannot know what would have occurred if the door had been kept closed: would the same processes have arisen without direct access to conscious experience? To what extent can we disentangle the neural basis of conscious perception *per se* from cognitive access mechanisms? Accessibility seems, thus, to be a constitutive part (though not causally related) of perceptual awareness, further increasing the level of complexity to assess and investigate the phenomenon of perception itself.

Another point is that subjective measure protocols are systematically and unavoidably influenced by the response criterion setting (van Gaal and Fahrenfort, 2008; Irvine, 2013), as already highlighted within both signal detection theory (Macmillan and Creelman, 1990) and drift-diffusion model frameworks (White et al., 2012). Decision-related aspects represent a bridge between sensory and motor processes necessary to perform a discrimination task. The discrimination process would, indeed, be the result of subprocesses, such as a sensory process that encodes the physical stimulation and a decision process that determines the response. Within this perspective, the behavioral performance is characterized by the sensitivity (how well the participants perceive the stimulus) and the criterion (how the participants choose to respond). Since these two factors are potentially independent, the criterion can be experimentally shifted without affecting the perceptual sensitivity. Specifically, when presenting near-threshold stimuli where participants are required to report trial-by-trial if they were aware of them, an internal criterion is set; according to this criterion, a given threshold of accumulated sensory evidence is needed to prompt an “aware response” whereas below this level, an “unaware rating” is given. From, a behavioral point of view, this threshold can be shifted, leading to a liberal criterion, which is characterized by a higher amount of aware responses (increased hit trials) and minimized missed detections, or to a conservative criterion, which is, instead, associated with a reduction of aware responses (decreased hit trials) and an increased exposure to false alarms (Lynn and Barrett, 2014; Kloosterman et al., 2019). Such pattern results even more exacerbated in the presence of perceptual uncertainty due to weak sensory stimuli, as in the case of contrastive analysis with stimuli at the threshold. The criterion is naturally different across participants as a function of several factors, including personality traits, cognitive styles, and stimulus expectation (Kantner and Lindsay, 2012; Bang and Rahnev, 2017). Moreover, a significant shift in criterion can be driven by the experimental design as well (e.g., amount of catch trials, visibility of the target, task instructions or asymmetries in stimulus-response reward, etc.) since the decision strategy is flexibly tailored depending on the experimental context (Lynn and Barrett, 2014; Bang and Rahnev, 2017; Kloosterman et al., 2019). The response criterion has also been of substantial interest within parallel lines of research regarding recognition memory (Hockley, 2011) and decision making (Gold and Shadlen, 2001; Wyart and Tallon-Baudry, 2009; Kloosterman et al., 2019), where analogous simple perceptual decision tasks requiring subjective reports are commonly employed. In this respect, however, the temporal relation between the subjective perceptual awareness of the experimental stimuli and the decision completion has only poorly been explored.

So far, no converging evidence has unveiled the temporal dynamics of the groundbreaking taxonomy put forward by Aru et al. (2012): the exact temporal windows of pre-conscious and post-perceptual processes should still be clearly identified, leaving open the intense debate about the best ERP component, among those that correlate to awareness (i.e., VAN and LP), representing the proper NCCs. In addition, it remains unclear whether preceding and following processes are fully dissociated from perceptual experience. Within this fragmented framework of perspectives, we tried to turn the aforementioned weakness of the contrastive analysis to our advantage. Since this approach might not isolate the solely NCCs due to contaminations from other cognitive processes characterizing the aware condition only, we reasoned that we could intentionally modulate a possible source of contamination instead of focusing on avoiding it. In so doing, we held constant everything else apart from the response criterion, which is known to affect how a percept is converted into decision evidence, and it is thereby involved in a post-perceptual stage (Green and Swets, 1966; White and Poldrack, 2014). The resulting modulation should affect the ERP component(s) not exclusively reflecting the conscious perception itself. This manipulation can provide a clearer picture of NCCs, which can be disentangled from their consequences and shed novel light on the close relationship between electrophysiological correlates of awareness and sensory evidence accumulation leading to the decision stage.

## Experiment 1

### Materials and Methods

#### Participants

**Figure 1:**
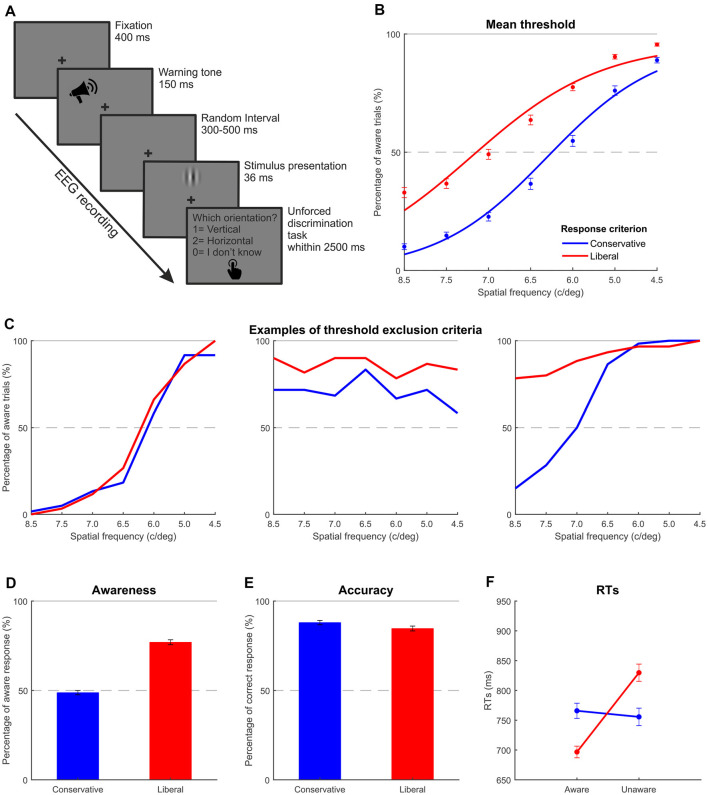
Experiment 1: thresholding and behavioral results. **(A)** Trial structure of the discrimination task carried out in Experiments 1 and 2. **(B)** Mean threshold functions for the two response criteria. The blue line represents the fitted function obtained adopting a conservative criterion, while the red line shows the fitted function obtained adopting a liberal criterion. Each dot indicates the mean performance across the participants included in the experimental sample corresponding to each Gabor spatial frequency. **(C)** Three possible examples of participant exclusion criteria through the thresholding phase. On the left, the two functions are overlapped indicating a failing modulation of the response criterion. In the middle, one of the two experimental conditions elicited a flat pattern and highlighted about the same awareness rate regardless of the spatial frequency. On the right, the whole psychometric function of one of the two experimental conditions is above the 50% level, making impossible to determine the threshold value. **(D,E)** Average awareness and accuracy rates as a function of response criterion. **(F)** Average reaction times per awareness and response criterion. Error bars represent standard errors of the mean (SEM).

Fifty-nine right-handed participants with normal or corrected-to-normal vision were recruited for the study. After the initial threshold assessment performed in order to select the stimuli to be used in the main EEG experiment (see “Experimental Procedure” section for details), 21 participants were discarded: six failed to modulate the response criterion (conservative | liberal) as required in the task (i.e., showing overlapped psychometric functions, e.g., Figure 1C on the left). A total of 12 showed the percentage of the aware trials was not modulated across levels of stimulation (i.e., flat psychometric function in at least one of the two experimental conditions, e.g., Figure 1C). Three were discarded because 50% threshold value could not be determined in at least one condition since all the stimuli reached higher aware trial percentages (i.e., the entire psychometric function above the 50% level, e.g., Figure 1C on the right). Additionally, data from four participants were excluded from the EEG analysis due to either technical issues during recording or incomplete data acquisition. Of the remaining 34 participants, one was further excluded because he adopted the opposite response bias shift, and nine were also further excluded because the liberal-conservative difference in awareness was less than 10%. Hence, the final sample comprised 24 participants (13 females, mean age = 21.83, SD = 1.61) who were naïve as to the purpose of the study. It is worth noting that such a high participant exclusion rate is not unusual in this kind of experiment (for examples see Wilenius and Revonsuo, 2007; Salti et al., 2012; Tagliabue et al., 2016; Travis et al., 2019; Ye et al., 2019).

All the participants were recruited from the student community of the University of Verona and were paid for their participation. Written informed consent was obtained from all participants prior to study enrollment. The experimental procedure was approved by the local ethics committee and conducted in accordance with the 2013 Declaration of Helsinki.

#### Apparatus and Stimuli

Visual stimuli consisted of vertically or horizontally oriented sinusoidal Gabor patches of about 4° of visual angle (Michelson contrast 0.50). Spatial frequencies employed in the experiment were individually calibrated by means of a threshold procedure performed prior to the main experiment. During this phase, seven different preselected vertical and horizontal spatial frequencies (4.5, 5.0, 6.0, 6.5, 7.0, 7.5, and 8.5 c/deg) were presented to the participants. In catch trials, the stimulus consisted of a patch characterized by a plaid pattern resulting from the overlap of two orthogonally oriented Gabor patches with a spatial frequency of 4.5 c/deg.

Stimuli appeared for 36 ms at the top center of the screen (11° above the fixation cross along the vertical meridian) against a gray background (3.9 cd/m^2^). A 17-inch CRT monitor (LG L1753HM, resolution 1,024 × 768 pixels, refresh rate 85 Hz) placed 57 cm away from the observer’s head was used to display the stimuli which were previously generated by MATLAB custom code (The Mathworks Inc., Natick, MA, USA). E-prime 2.0 software (E-Prime Psychology Software Tools Inc., Pittsburgh, PA, USA) running on a Windows PC was used both to present stimuli and to collect behavioral data. Before starting the data collection, the timing of the event markers and the effective duration of the stimuli were verified by means of a photodiode and an oscilloscope.

#### Experimental Procedure

Experiment 1 was designed to manipulate participants’ response criterion in a simple perceptual discrimination task by inducing a conservative or liberal bias through different verbal instructions given prior to the experiment. In the conservative session, participants were instructed to report the Gabor orientation only if they clearly perceived it, while, in the liberal session, participants were asked to report the orientation of the stimulus whenever they had a minimal impression. Each participant consecutively completed the two phases of the experiment (threshold assessment and EEG experiment) on the same day. In total, the entire experimental procedure lasted about 4 h including the set-up of the EEG cap and short breaks between blocks.

##### Thresholding

First, each participant underwent a behavioral threshold assessment procedure with the aim of selecting individually calibrated spatial frequencies (one for horizontal and one for vertical Gabor patches) for the main experimental session. The threshold was estimated using the method of constant stimuli with a three-alternative unforced-choice task (i.e., the possible responses were represented by the two orientations and the additional option “do not know”; Kaernbach, 2001). According to this method, a set of vertical and a set of horizontal spatial frequencies were randomly presented to the observers, and the observers were asked to report, *via* a button press, the orientation of the presented stimulus (vertical or horizontal, respectively keys 1 and 2) or that they could not discriminate it (“do not know” answer, key 0). This allowed participants to modulate their response criterion and, at the same time, to keep the working memory load as low as possible due to the employment of a single task.

Participants were seated in a dimly lit room with their head supported by a chinrest in order to keep the distance from the monitor constant and minimize head movements. Two separate sessions of threshold assessment were consecutively carried out, and they were identical except for the induced response criterion bias (conservative | liberal). The order of the two sessions was counterbalanced across participants. Each session included six blocks of 77 trials each for a total of 462 trials. Each (vertical or horizontal) spatial frequency was presented 30 times, while there were 42 catch trials. The trial sequence started with a central fixation cross followed by a 1,000 Hz warning tone. After a random interval (ranging from 300 to 500 ms) one of the preselected stimuli was displayed, and participants were asked to make a button press response using their right hand, after which no feedback was provided (see Figure 1A). At the end of the threshold procedure, one horizontal frequency value and one vertical frequency value, both yielding 50% of aware responses (i.e., all responses excluding the “do not know” choices, which represent unaware condition) were visually identified for each of the two sessions (conservative | liberal) on the basis of the obtained psychometric functions. Then, the mean between the conservative and liberal horizontal values and the mean between the conservative and liberal vertical values were calculated separately (intermediate levels of spatial frequency other than those tested in the threshold assessment procedure could also be chosen) and used as spatial frequency of the target stimuli employed during the subsequent main phase of the experiment so that, in each participant, the amount of aware responses could be comparable for horizontal and vertical Gabors.

##### Main Experiment

The main experiment consisted of two sessions with a short break of 15 min between them. As in the threshold assessment phase, the two sessions were identical, but a different response criterion shift was induced in the participants: in one session they were asked to adopt a conservative criterion while in the other one they were encouraged to use a liberal criterion. Again, the order of the two sessions was counterbalanced across participants. The task was the same performed in the threshold phase, where participants were requested to discriminate the orientation of a Gabor (Figure 1A). However, in this context, just two kinds of near-threshold vertical or horizontal Gabor patches (whose spatial frequency values were determined during the previous phase) were presented and the EEG signal was concomitantly recorded. Each experimental session—composed of six blocks of 77 trials—yielded a total of 462 trials: 210 horizontal Gabor patches, 210 vertical Gabor patches, and 42 catch trials (10% of critical trials).

#### EEG Recording and Pre-processing

A continuous EEG signal was recorded through a BrainAmp system (Brain Products GmbH, Munich, Germany–Brain Vision Recorder) using a Fast’n Easy cap (EasyCap, GmbH, Herrshing, Germany) with 59 Ag/AgCl electrodes arranged according to the 10-10 International System. Additional electrodes were placed around the eyes (left and right canthi and above and below the right eye) for monitoring the electro-oculogram activity (EOG, blinks, and saccades in particular), while electrode AFz served as the ground and the right mastoid (RM) as the online reference. Electrode impedance was constantly kept below 5 KΩ throughout the experiment. Data were recorded at a sampling rate of 1,000 Hz with a time constant of 10 s as a low cut-off and a high cut-off of 250 Hz.

The EEG signal was first imported to the EEGLAB toolbox (v2019_0, Swartz Center for Computational Neuroscience, University of California at San Diego, Delorme and Makeig, 2004), resampled to 250 Hz, and high-pass filtered at 0.1 Hz. Power line noise (50 Hz and its harmonics) was attenuated by means of adaptive multitaper regression implemented in the CleanLine EEGLAB plugin. All scalp channels were then offline re-referenced to the mean of the two mastoids (RM-LM) prior to data segmentation into 3-s epochs (from −1,000 ms to 2,000 ms with respect to the stimulus onset). An independent component analysis (ICA) using the extended InfoMax algorithm (Bell and Sejnowski, 1995) was performed. The 63 resulting independent components were visually inspected and removed when identified as artifactual due to blinks, eye movements, and muscle activity. Subsequently, a low-pass filter at 40 Hz was applied. The epoch window was shortened to 1,300 ms (starting from 300 ms before the stimulus), and, thereafter, baseline correction was performed based on the pre-stimulus period. Before averaging, the epochs showing activity contaminated by extreme values (±75 μV) in any of the 59 electrodes were automatically rejected (on average, 13% of the epochs were discarded). Finally, stimulus-locked grand-average for aware (trials in which the participants reported to have discriminated the stimulus) and unaware (“do not know” responses) critical trials were computed separately for each response criterion bias (conservative and liberal).

#### Data Analysis

##### Behavioral Data

Data were processed using MATLAB 2019a and analyzed with IBM SPSS Statistics for Windows, version 22. For each participant, trials with reaction times lower than 150 ms and higher than 1,500 ms, as well as trials with no response, were not included in the analyses (about 3% of the data). Horizontal and vertical trials were systematically collapsed for (behavioral and EEG) data analysis. Paired-samples *t*-test was applied to compare mean percentages of aware responses for conservative criterion with those for liberal criterion. The same statistical test was used to assess whether the mean percentages of correct responses were significantly different between the two response criteria. Reaction times were submitted to a repeated-measures ANOVA with awareness (Aware | Unaware) and criterion (Conservative | Liberal) as within-subject factors.

##### EEG Data

Time windows and electrodes chosen for statistical analyses were selected both according to previous literature (Koivisto and Revonsuo, 2010; Tagliabue et al., 2016) and on the basis of visual inspection of the grand-average ERPs (i.e., the electrodes with the highest peak amplitude in the component of interest when looking at the difference waves between conditions).

Grand-average ERPs were submitted to repeated-measure ANOVA with Awareness and Criterion as within-subject factors. The main effects were computed using parametric statistical routines with a statistical threshold of 0.05 implemented in EEGLAB study, while the Mass Univariate ERP Toolbox (Groppe et al., 2011) was used to further explore the interactions.

### Results

#### Behavioral Results

##### Thresholding

As expected, the awareness rate increased as the spatial frequency decreased, and it was overall higher in the liberal than in the conservative condition. When a catch stimulus was presented, on average, participants chose the “do not know” response category 86.00% of the time, as they could not fittingly discriminate the orientation. Moreover, no differences were found between the two criteria (conservative catch 86.93% vs. liberal catch 85.06%; *t*_(23)_ = 0.594; *p* = 0.594).

At the end of the threshold procedure, which aimed at individually selecting the stimulus yielding 50% of aware responses in both the conditions (conservative and liberal) and in both the orientation (vertical and horizontal), the mean spatial frequency chosen was 6.9 c/deg for vertical stimuli and 6.2 c/deg for horizontal stimuli. Mean psychometric functions for the two response criteria, computed by collapsing data across stimulus orientation (vertical and horizontal), are shown in Figure 1B. For illustrative purposes only, data averaged across participants were fitted with a logistic psychometric function (lapse rate 4%) with a maximum likelihood criterion using Palamedes toolbox for Matlab^1^ (Mazzi et al., 2017a,b).

##### Main Experiment

On average, participants reported a higher awareness rate (computed taking into consideration responses “vertical” and “horizontal” of the three-alternative choices over the total amount of target stimuli) in the liberal session (76.96%) than in the conservative session (48.78%; *t*_(23)_ = −11.729; *p* < 0.001), indicating that participants performed the task as instructed and could modulate their response bias (Figure 1D). Around 96% of the time, catch stimuli were correctly categorized as unaware with respect to the orientation, thereby indicating the reliability of performance. No differences were found between the two criteria (conservative catch 94.79% vs. liberal catch 96.90%; *t*_(23)_ = −1.529; *p* = 0.140). Participants were significantly more accurate in the conservative session (87.97%) than in the liberal session (84.65%; Figure 1E; *t*_(23)_ = 2.336; *p* < 0.05). Due to the low amount of catch trials representing only 10% of target stimuli and their easily detectable nature (low spatial frequency plaid pattern) resulting in a low false alarm rate, signal-detection measures such as sensitivity and criterion (Green and Swets, 1966) were not computed.

The two-way repeated-measures ANOVA conducted on reaction time data with awareness and criterion as within-subject factors revealed a main effect of awareness (*F*_(1,23)_ = 7.912; *p* < 0.05, $\eta_{\text{p}}^{2}$ = 0.26): aware trials (731 ms) were faster than unaware trials (792 ms; Figure 1F), while the main effect of criterion was not found to be significant (761 ms for conservative vs. 763 ms for liberal condition; *F*_(1,23)_ = 0.039; *p* = 0.845, $\eta_{\text{p}}^{2}$ = 0.00). Importantly, the interaction between awareness and criterion was significant (*F*_(1,23)_ = 34.159; *p* < 0.001, $\eta_{\text{p}}^{2}$ = 0.60), highlighting that awareness differently affects the two criteria: while conservative criterion RTs for aware trials did not differ from RTs of trials classified as unaware in the same condition (766 ms conservative aware vs. 756 ms conservative unaware; *t*_(23)_ = 0.419; *p* = 0.679), a significant difference was reported pairwise comparing aware and unaware RTs of the liberal condition (*t*_(23)_ = −5.170; *p* < 0.001) with shorter RTs for aware trials (follow-up *t*-tests with Bonferroni correction applied).

##### EEG Results

Across participants, the mean trial number per condition considered in the analyses after EEG pre-processing was as follows: 197 for conservative aware, 209 for conservative unaware, 304 for liberal aware, and 94 for liberal unaware.

**Figure 2:**
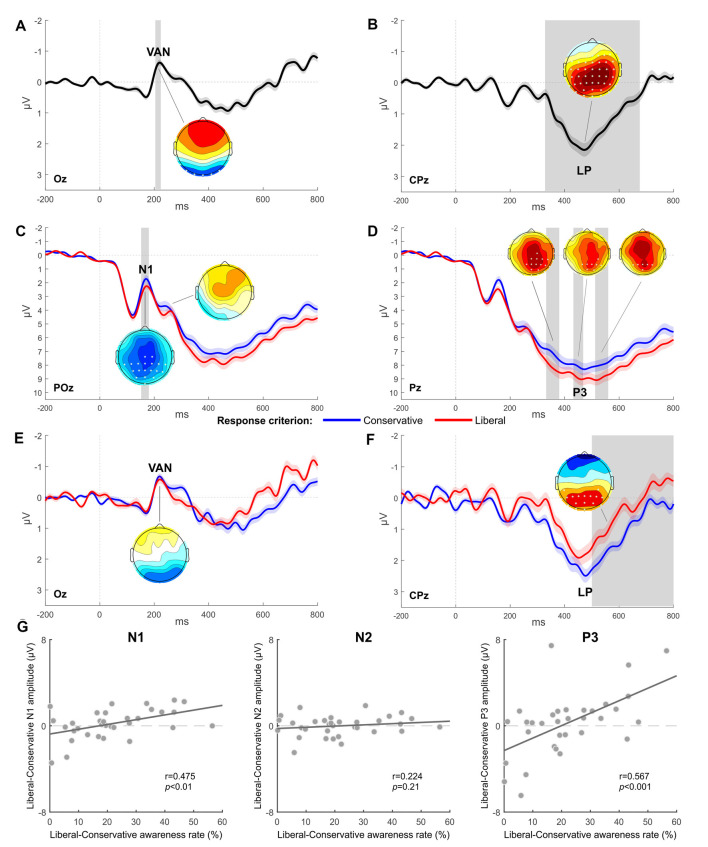
Experiment 1: EEG results. **(A,B)** Grand-average waveform computed as the difference between the aware and unaware condition and respectively highlighting a significant Visual Awareness Negativity (VAN; electrode Oz) followed by a significant Late Positivity (LP; electrode Pz). **(C,D)** Grand-average of ERPs obtained for each response criterion (electrode POz for N1 and N2 components, electrode Pz for P3 component). **(E,F)** Grand-average difference waves (aware minus unaware condition) obtained for each response criterion (electrode Oz for VAN, electrode CPz for LP). The most representative electrodes are respectively chosen for each ERP component. The shaded area of the waveforms represents SEM at each time point. Significant time windows are vertically highlighted in gray. Scalp distribution maps represent the voltage difference between conditions, with asterisks indicating statistically significant electrodes (*p* < 0.05) in the given contrast and time window. The amplitude scale of topographical maps is set at ±1 μV. **(G)** Scatter plots showing Pearson correlations between the magnitude of response criterion shift (represented on the x-axis) and the difference in amplitude between the two response criterion conditions for respectively N1, N2, or P3 components (represented on the y-axis). Each circle represents a participant.

Consistent with previous studies (e.g., Koivisto and Revonsuo, 2010), a significant main effect of Awareness was found for VAN and LP, respectively, in the 204–224 time window (electrodes O1, Oz, O2, PO7, PO8, and P7, all *p*s < 0.05) and in the 328–676 time window (all electrodes except for Fp1, Fp2, AF7, AF3, AF8, F7, F5, F3, and FT7, all *p*s < 0.05). Figures 2A,B show the difference waves computed by subtracting the unaware from the aware condition and highlights an early negative deflection (VAN) at posterior temporal electrodes (Oz in Figure 2A) followed by a positive enhancement at centro-parietal sites (CPz in Figure 2B).

A main effect of Criterion was also found to be significant. In particular, N1 showed a greater amplitude in the 152–180 time window (in electrodes Cz, C2, CP5, CP3, CP1, CPz, CP2, CP4, CP6, P3, P1, Pz, P2, P4, P6, PO3, POz, PO4, and Oz, all *p*s < 0.05) when participants were requested to adopt a conservative criterion compared to the session adopting the liberal criterion (Figure 2C). The P3 component was also significantly modulated as a function of the criterion adopted: the liberal session elicited, indeed, a greater amplitude respect to the conservative session in the time windows 332–380 ms (electrodes C2, T8, CPz, CP2, CP4, CP6, TP8, P1, Pz, P2, P4, P6, P8, POz, PO4, PO8, Oz, and O2, all *p*s < 0.05), 432–468 ms (electrodes CP4, P1, Pz, P2, P4, P6, PO3, POz, and PO4, all *p*s < 0.05), and 512–560 ms (electrodes P3, P1, Pz, P2, P4, and POz, all *p*s < 0.05; Figure 2D). There was no evidence of a significant amplitude difference at the N2 latency.

Critically, a significant interaction between the factors Awareness and Criterion was found for LP in the 500–800 time windows in electrodes O1, Oz, PO7, PO3, POz, PO4, PO8, P7, P5, P3, P1, Pz, P2, P4, P6, CP5, and CP4 (all *p*s < 0.05), with a more pronounced LP in the conservative condition than in the liberal one (Figure 2F). The interaction was not significant for VAN (Figure 2E).

To further explore the effect of response criterion shift on ERP components, we conducted Pearson’s correlation analysis between the individual behavioral difference of awareness rate (liberal minus conservative) in order to have a measure of how much each participant could shift her/his response criterion, and the difference in amplitude (liberal minus conservative) of each ERP component considered in the previous ANOVA. The aim of this analysis was to determine whether participants who could modulate to a greater extent their response criterion, also showed increased ERP differences in the different components’ amplitude. Correlations were conducted on the entire sample (*N* = 33), including the nine participants whose liberal-conservative difference in awareness was less than 10% and thus excluded from the ERPs analysis. The maximum peak latency of N1, N2, and P3 components was respectively identified from the grand-average of all trials (liberal and conservative sessions collapsed) in three different electrodes (PO3 for N1, PO8 for N2, and Pz for P3) and used to extract the corresponding individual amplitudes. N1 (*r* = 0.475, *p* < 0.01) and P3 (*r* = 0.567, *p* < 0.001) components positively correlated with criterion, while N2 did not show any correlation effect (*r* = 0.224, *p* = 0.21; Figure 2G). Crucially, the same correlation analysis was performed for all the critical electrodes according to the specific topography of each component. Results were always consistent with those reported above: N1 and P3 components remained significant in all cases, while N2 never reached the significance threshold.

### Experiment 2

In Experiment 1, we manipulated the participant’s response criterion by inducing either a liberal or a conservative bias in two different experimental sessions. Results showed no evidence for a difference between the two conditions in the N2 window, which is thought to trigger the process of sensory evidence accumulation (Loughnane et al., 2016). Building upon this observation, in Experiment 2, we sought to further explore dynamics underlying visual awareness by asking participants to follow their natural response criterion with the aim of comparing behavioral and electrophysiological data from the two experiments.

#### Materials and Methods

##### Participants

Sixteen out of 24 participants who were included in Experiment 1 (nine females, mean age = 21.88, SD = 1.50) agreed to perform an additional experimental session and were paid for their participation.

##### Stimuli, Apparatus, and Experimental Procedure

The stimuli, apparatus, and task were the same as in Experiment 1 except that participants completed one session only (for a total of 462 trials) in which they were asked to adopt their own natural response criterion (hereafter called “Own criterion session”). Importantly, the Gabor spatial frequencies employed were the same as in the previous sessions (i.e., those chosen individually by means of the threshold assessment in Experiment 1) so that comparison among sessions could be performed. The session lasted about 1.5 h including the EEG cap set-up and short breaks when needed.

##### Data Analysis

Since EEG recording settings and data pre-processing were the same as in Experiment 1, both behavioral and EEG data from the own criterion session were compared with those of the two previous sessions (conservative and liberal), considering selectively the participants who also performed Experiment 2. Specifically, awareness and accuracy percentages collected in Experiment 2 (own session) were compared to conservative and liberal sessions by means of two one-way ANOVAs at three levels (i.e., conservative, liberal, and own). Moreover, a 2 × 3 repeated measures ANOVA assessed the effect of the different criteria on the awareness in terms of reaction times. As *post hoc* analysis, multiple paired *t*-test with Bonferroni correction was applied. Moreover, grand average ERP waveforms were submitted to a two-way repeated-measure ANOVA with Criterion (Conservative, Liberal, and Own) and Awareness (Aware and Unaware) as within-subject factors. The Greenhouse-Geisser correction was applied where data violated the sphericity assumption. As in Experiment 1, time windows and electrodes for the analysis were chosen based on the inspection of grand-averaged ERPs across conditions.

#### Results

##### Behavioral Results

**Figure 3:**
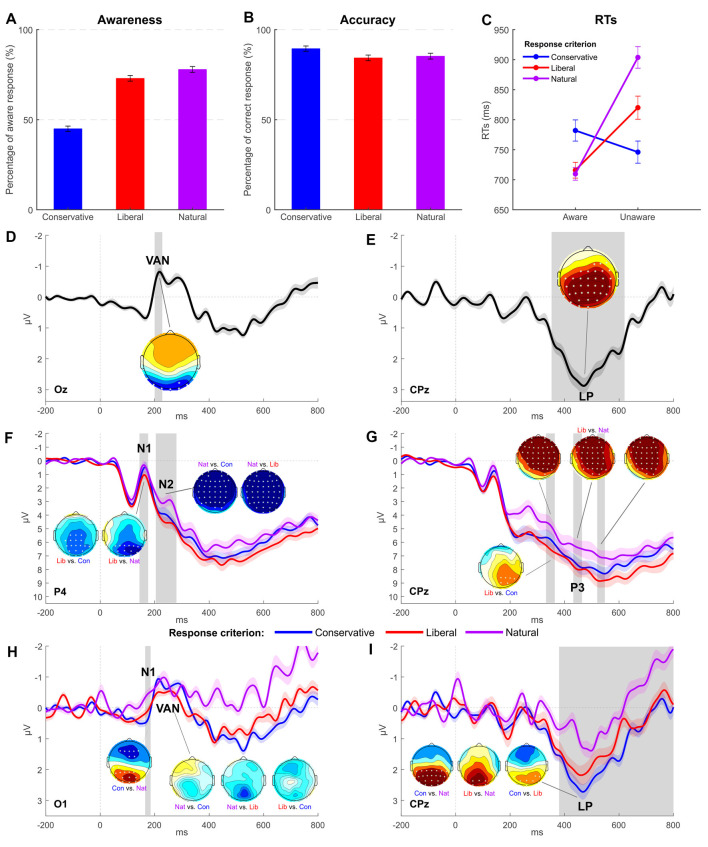
Experiment 2: behavioral and EEG results. **(A,B)** Average awareness and accuracy rates across the three conditions of interest. **(C)** Average reaction times per awareness and response criterion. Error bars represent SEM. **(D,E)** Grand-average waveform computed as the difference between the aware and unaware condition and respectively highlighting a significant VAN (electrode Oz) followed by a significant LP (electrode CPz). **(F,G)** Grand-average of ERPs obtained for each response criterion (electrode P4 for N1 and N2 components, electrode CPz for P3 component). **(H,I)** Grand-average difference waves (aware minus unaware condition) obtained for each response criterion (electrode Oz for VAN, electrode CPz for LP). The most representative electrodes are respectively chosen for each ERP component. The shaded area of the waveforms represents SEM at each time point. Significant time windows are vertically highlighted in gray. Scalp distribution maps represent the voltage difference between conditions, with asterisks indicating statistically significant electrodes (*p* < 0.05) in the given contrast and time window. The amplitude scale of topographical maps is set at ±1 μV.

For the natural criterion session, 77.92% of the 420 critical trials was rated as aware. The one-way ANOVA with Criterion as factor (with three levels: conservative, liberal, and own) disclosed a significant difference across sessions adopting different response criteria (*F*_(2,30)_ = 44.557; *p* < 0.001, $\eta_{\text{p}}^{2}$ = 0.75; see Figure 3A). Pairwise comparisons (Bonferroni correction applied) indicated a lower awareness rate for the conservative session (44.97%) compared both to the liberal (72.95%, *p* < 0.001) and own (*p* < 0.001). On average, participants reported to be unaware of catch trial orientation (plaid pattern) in 95.37% of the cases, and no differences were revealed across criteria (conservative = 93.52%, liberal = 95.95%; *F*_(1.098,16.475)_ = 1.119; *p* = 0.312, $\eta_{\text{p}}^{2}$ = 0.07). As with accuracy data (own = 85.27%), we found a main effect of criterion (*F*_(1.447,21.706)_ = 6.686; *p* < 0.05, $\eta_{\text{p}}^{2}$ = 0.31) where the only significant pairwise comparison indicated the conservative criterion accuracy (89.46%) as higher than liberal (84.31%, *p* < 0.001; Figure 3B).

To further examine the relationship between awareness and criterion, RTs were analyzed with a two-way repeated measures ANOVA (Figure 3C) that revealed a significant effect of Awareness (*F*_(1,15)_ = 21.879; *p* < 0.001, $\eta_{\text{p}}^{2}$ = 0.59) with aware RTs (735 ms) overall shorter than unaware (823 ms). No differential RT modulations were found across criteria (*F*_(2,30)_ = 2.773; *p* = 0.078, $\eta_{\text{p}}^{2}$ = 0.16; conservative = 764 ms, liberal = 768 ms, own = 807 ms). The Awareness by Criterion interaction was statistically significant (*F*_(2,30)_ = 18.222; *p* < 0.001, $\eta_{\text{p}}^{2}$ = 0.55). *Post hoc* pairwise comparisons were then carried out with the aim of exploring this interaction. According to the Bonferroni multiple testing correction, the nine possible comparisons were considered to be significant if *p* ≤ 0.0055 (0.05/9). In the conservative session, RTs of aware trials were not different from those of unaware trials (782 ms vs. 746 ms, respectively; *t*_(15)_ = 1.285; *p* = 0.218). By contrast, in liberal (*t*_(15)_ = −3.674; *p* < 0.01; 716 ms vs. 820 ms) and own (*t*_(15)_ = −6.349; *p* < 0.001; 710 ms vs. 904 ms) sessions, aware trials were shorter than unaware, thereby confirming that participants reacted differently as a function of awareness when adopting the different response criteria. Comparing the three criteria either within aware or unaware responses, the only significant contrasts were the ones comparing conservative to liberal unaware trials (*t*_(15)_ = −3.651; *p* < 0.01; 746 ms vs. 820 ms) and conservative to natural unaware trials (*t*_(15)_ = −4.510; *p* < 0.001; 746 ms vs. 904 ms), while no differences were detected between liberal and natural unaware trials (*t*_(15)_ = −2.572; *p* = 0.021; 820 ms vs. 904 ms) and within aware trials [Con. Aw. vs. Lib. Aw. (*t*_(15)_ = −2.741; *p* = 0.015; 782 ms vs. 716 ms), Con. Aw. – Nat. Aw. (*t*_(15)_ = −2.546; *p* = 0.022; 782 ms vs. 710 ms) and Lib. Aw. – Nat. Aw. (*t*_(15)_ = 0.263; *p* = 0.796; 716 ms vs. 710 ms)].

##### EEG Results

After artifact rejection, the mean number of epochs used for statistical analyses was 183 for conservative aware, 223 for conservative unaware, 288 for liberal aware, 109 for liberal unaware, 314 for own aware, and 93 for own unaware condition.

A main effect of awareness was observed for VAN and LP. In particular, VAN was significant between 200 and 228 ms at posterior electrodes (O1, Oz, O2, PO7, PO4, PO8, P7, and P8, all *p*s < 0.05; Figure 3D), while LP was significant between 352 and 620 ms at centro-parietal sites (all electrodes except for Fp1, Fp2, AF7, AF3, AF4, AF8, F7, F5, F3, F1, Fz, FT7, FC5, FC3, O2, PO8, and P8, all *p*s < 0.05; Figure 3E).

Furthermore, the analysis revealed a main effect of criterion for components N1, N2, and P3. A significant N1 modulation was found in the 144–176 time window (Figure 3F), and follow-up comparisons showed a greater amplitude for own and conservative conditions as respect to the liberal condition in the following electrodes: P3, P1, Pz, P2, P4, P6, P8, PO3, POz, PO4, O1, Oz, O2, and PO8 (all *p*s < 0.05). Differently from Experiment 1, the N2 component was found to be significant in the 204–280 time window in all the electrodes except for F8, FT7, FC6, FT8, T7, T8, TP7, TP8, P7, P8, and PO7 (Figure 3F). Follow-up paired *t*-tests showed a clear amplitude enhancement of the N2 component for own session trials, while conservative and liberal did not differ from each other.

P3 component was significant in several time windows: 332–364 ms (all electrodes *p*s < 0.05 except for FC6, FT8, T7, C6, T8, TP7, CP5, CP6, TP8, P7, P5, P3, Pz, P8, PO7, PO3, POz, PO8, O1, Oz, and O2), 432–464 ms (electrodes FP1, FP2, AF7, AF3, AF4, AF8, F5, F3, F1, Fz, F2, FC3, FC1, FCz, FC2, C3, C1, Cz, C2, CP3, CP1, CPz, P3, P1, POz, and PO4), and 520–548 ms (significant electrodes FP1, FP2, AF7, AF3, F5, F1, Fz, F2, FC3, FC1, FCz, FC2, C5, C3, C1, Cz, C2, CP3, CP1, and CPz; Figure 3G). Follow-up *t*-tests revealed that the liberal criterion session elicited the greatest P3 in amplitude compared to conservative and own sessions, which were not different from each other.

The two-way interaction was significant for LP in the 380–800 time window in electrodes O1, Oz, O2, PO7, PO3, POz, PO4, PO8, P7, P5, P3, P1, Pz, P2, P4, P6, P8, CP5, CP3, CP1, and CPz (all *p*s < 0.05; Figure 3I). *Post hoc* two-tailed paired *t*-tests revealed that LP was significantly more pronounced in conservative condition than in liberal and own conditions. A significant difference was found also between liberal and own conditions.

The interaction resulted to be significant also for the N1 component in the 165–185 time window (electrodes O1, Oz, O2, PO7, PO3, POz, PO4, and P5). *Post hoc* two-tailed paired *t-tests* revealed that N1 was significantly greater in amplitude in the conservative condition when compared to the own condition (Figure 3H).

## Discussion

The present article sought to dissociate the electrophysiological NCCs from potential post-perceptual confounds. Indeed, as claimed by Aru et al. (2012), contrastive analysis results would usually overestimate NCCs in report-based paradigms by including processes not strictly corresponding to the proper NCCs. Our manipulation intended to induce a response bias shift through different task instructions, in order to probe, from an electrophysiological point of view, how conscious perception interacts with decision processes to identify which ERP component cannot reflect perception *per se* since it is modulated by such post-sensory decision processes.

Behavioral results revealed that participants could successfully adopt the expected response criterion across conditions, as shown by a reduced awareness rate along with increased accuracy for the conservative criterion. This confirms that participants were less likely to report the stimulus orientation in the conservative session as compared to the liberal session. Reaction times were modulated as well, showing a different pattern as a function of awareness when adopting the conservative criterion compared to the other sessions: while in both liberal and natural sessions participants reacted faster to aware than unaware stimuli, no differences were found within the conservative session. We can speculate that the speed-up of unaware responses could be due to the adopted conservative criterion since participants were encouraged to choose the unaware option when not clearly perceiving the orientation of a stimulus at threshold. Moreover, they may have inhibited aware responses even though they were aware of the stimuli. Overall, no behavioral differences were found between liberal and natural criteria. This might depend on the spontaneous participants’ tendency to try to be as accurate as possible rather than “accurately biased.” This therefore relies on sensory information instead of adopting the expected decision rule (Kantner et al., 2015). From this point of view, implementing a conservative strategy may be easier than adopting a liberal criterion where participants are more likely to be inaccurate since aware answers are driven by minimal perceptual impressions. Moreover, the high perceptual uncertainty caused by the presentation of near-threshold stimuli could have also played a role in this process, making participants less prone to guess the stimuli orientation and thus reducing the magnitude of liberal bias shift if compared to natural session. Nevertheless, the lack of a clear behavioral shift for the liberal session in comparison with the natural criterion session does not preclude that electrophysiological modulations have occurred.

As to the electrophysiological effect of response criterion manipulation regardless of the awareness level, the main findings were the decrease of N1 and, conversely, the increase of P3 amplitude for the liberal condition over (parieto-occipital electrodes for the former and over centro-posterior electrodes for the latter. Importantly, the more the participants shifted their criterion across sessions, the bigger was the difference in amplitude of the modulated components, and this suggests that neural mechanisms underlying decision making occurred in this time window. In keeping with that, P3 has been suggested to resemble the Centro-Parietal Positivity (CPP) by showing comparable latency and topography (O’Connell et al., 2012; Kelly and O’Connell, 2013; Twomey et al., 2015). This prominent component has been found to correlate with the accumulation of sensory evidence and is modulated by the timing and the accuracy of the subjective report, thereby tracing the evolving neural dynamics leading to perceptual decisions. Indeed, according to the literature on decision-making, simple perceptual decisions, such as those requested in our paradigm, are conceptualized as the result of sequential processing stages ensuring the translation of task-related physical stimulation into a behavioral decision (Sternberg, 1969). Specifically, the three steps would reflect functionally distinct processes: the sensory encoding phase, where the physical stimulus is encoded by the visual cortex; the decision formation (i.e., CPP), which is based on evidence accumulation over time until a decision threshold is reached; and, finally, the corresponding motor planning and execution. Unfortunately, to date, the temporal dimension of these steps remains still poorly explored (but see Mostert et al., 2016).

With regard to N1, it is commonly thought to be involved in early sensory and perceptual processing, as well as in the orienting of attention toward task-relevant stimuli (Luck et al., 1990; Posner and Dehaene, 1994; Hillyard et al., 1998). Given that participants did not shift their response criterion on a trial-by-trial basis but across sessions, one possibility is that the N1 decrease associated with the liberal criterion reflects an inhibitory effect of perceptual processes due to a sort of task-set “less engaging” from an attentional point of view, which is implemented to facilitate the task performance. Indeed, in the liberal session, a higher response rate is requested *a priori*, independently from accuracy. An alternative interpretative hypothesis, which does not exclude the former one, can be represented by the confidence degree: in a previous study investigating NCCs (Ye et al., 2019), N1 was modulated by the confidence level, showing an enhanced amplitude in correspondence to high-confidence trials when compared to slight-confidence trials. In this context, it seems plausible to assume a poorer confidence level in the liberal criterion session because at least a portion of the answers is based on faint perceptual impressions.

Furthermore, a significant enhanced N2 amplitude was found for the natural criterion as compared to the experimentally biased conditions (conservative and liberal sessions), and this was irrespective of the awareness level. This finding appears to be consistent with previous evidence showing that N2 might play a role in processes preceding the accumulation of evidence, specifically devoted to selecting task-relevant sensory information and enhancing their relative processing (Loughnane et al., 2016). This was a crucial step in the natural criterion session since participants were asked to make orientation judgments without following any predetermined decision rule but relying solely on the sensory evidence even though this led to uncertainty. Intriguingly, the N2 amplitude in our dataset did not correlate with the magnitude of the criterion shift adopted by the participants, suggesting that it would be perceptual in nature rather than related to decision processing.

In keeping with ERP awareness literature employing the classical contrastive design, when considering data independently from the response criterion, the typical pattern is replicated where VAN and LP correlate with visual awareness. As previously mentioned, at present, there is no unanimous consensus on the functional significance of VAN and LP (e.g., Rutiku and Bachmann, 2017), and different theories have been developed in this respect. Mainly, they can be classified as “early” or “late” theories of visual awareness depending on the temporal window considered to be critical for the emergence of awareness. Some studies have identified VAN as the earliest neural marker of visual awareness, fueling the idea that LP is more related to further post-perceptual processing rather than to conscious perception *per se* (Koivisto and Revonsuo, 2003, 2010; Wilenius-Emet et al., 2004; Pitts et al., 2014; Koivisto and Grassini, 2016; Koivisto et al., 2016; Eklund and Wiens, 2018; Mazzi et al., 2019; Ye and Lyu, 2019). Other evidence flowing into the late theories of visual awareness, usually endorsing the global workspace theory as well, have indicated that LP, instead, could represent the ERP component giving rise to conscious perception (Sergent et al., 2005; Babiloni et al., 2006; Del Cul et al., 2007; Lamy et al., 2009; Dehaene and Changeux, 2011; Salti et al., 2012; Boncompte and Cosmelli, 2018). According to this point of view, VAN would reflect a pre-conscious stage. Besides, others pointed out a twofold contribution of VAN and LP in triggering perceptual experience (Rutiku et al., 2015, 2016; Tagliabue et al., 2016; Derda et al., 2019; Ye et al., 2019). Our approach has the added benefit of going beyond these findings, dissociating the processes that are not directly related to awareness and may act as confounds.

Interestingly, our main result is that the LP amplitude is modulated as a function of both awareness and response criterion, while VAN is unaffected by this interaction. This finding supports the idea that VAN tracks visual awareness, whereas LP does not represent a pure index of awareness, though it would be involved in post-perceptual stages of processing. This observation corroborates a growing body of results obtained employing an approach analogous to that used here, intentionally designed to rule out potential confounds associated with awareness experience. Pitts et al. (2014), by means of a modified inattentional blindness paradigm, orthogonally manipulated visual awareness and task relevance, including a condition in which participants were aware of the experimental stimulus, whereas the access of perceptual information for the subjective report was not needed. Results showed that VAN was consistently observed regardless of the task-relevance of the stimuli, whereas P3b (i.e., LP) did not show the same pattern since it was absent when the stimuli were irrelevant to the task. This effect confirmed our findings suggesting that LP is crucial for reporting requirements rather than indexing the conscious perception itself. Similar conclusions were also drawn by Koivisto et al. (2016) in a study comparing different response requirements and participants were asked to report subjective experience in the GO condition, while they had to withhold responding in the NO-GO condition. Most recently, LP has been reported to be also modulated as a function of the temporal window in which the subjective report is requested (i.e., right after the stimulus presentation or after a 2 s delay; Ye and Lyu, 2019). In this case, even though the core finding does not differ from the two previously reported studies, the effect seems to be ascribable to working memory aspects since, in the delayed condition, perceptual decisions should be kept in mind.

In accordance with our findings indicating that LP reflects post-perceptual processing, it has been also suggested that LP is related to decision making, arguing that it is in many ways similar to CPP (Koivisto et al., 2016; Tagliabue et al., 2019). Indeed, it has recently been shown that CPP is mainly modulated by the perceived stimulus clarity rather than the physical stimulus intensity (Tagliabue et al., 2019). This evidence, on one hand, upholds the undeniable relationship between subjective perceptual experience and the evidence accumulation process (Wyart and Tallon-Baudry, 2009; Gregori-Grgič et al., 2011; de Lange et al., 2011). On the other hand, however, it further corroborates the idea that LP could not be strictly involved in the very early emergence of perceptual awareness but would reflect later stages.

Taken together, the present data along with previous evidence in literature support the idea that the modulation of the LP component is not exclusively driven by perceptual processes, but it is likely the result of a combination of different processes such as confidence and evidence accumulation that need to be disentangled with further data in order to pinpoint the corresponding neural signatures. Importantly, we can thus claim that LP is not causally involved in perceptual awareness but, instead, reflects also post-perceptual processes. For this reason, we endorse the view that there may be an earlier critical component responsible for the emergence of awareness. This could be represented by VAN, even though the account that it is an awareness prerequisite cannot be definitively ruled out at present. Moreover, we provided novel insight in disentangling perceptual awareness from decision making, especially from a temporal point of view.

## Data Availability Statement

The raw data supporting the conclusions of this article will be made available by the authors, without undue reservation, to any qualified researcher.

## Ethics Statement

The studies involving human participants were reviewed and approved by University of Verona. The patients/participants provided their written informed consent to participate in this study.

## Author Contributions

SS conceived the study. CM and SS contributed to the design of the experiment. GM carried out the data acquisition. CM and GM analyzed the data and performed the statistical analysis. CM and SS discussed the results and their interpretation. CM wrote the manuscript and SS provided critical revisions. All authors read and approved the submitted version.

## Conflict of Interest

The authors declare that the research was conducted in the absence of any commercial or financial relationships that could be construed as a potential conflict of interest.

## Footnotes

^1^http://www.palamedestoolbox.org
